# Supplementary material for: Tempo and mode in karyotype evolution revealed by a probabilistic model incorporating both chromosome number and morphology
Source: PLoS Genet. 2021 Apr 16;17(4):e1009502. doi: 10.1371/journal.pgen.1009502 (PMC8081341; doi:10.1371/journal.pgen.1009502)
Supplement: S1 Appendix — (DOCX) [file pgen.1009502.s001.docx]

**S1 Appendix**

**Stationary distribution of Markov process of karyotype evolution**

Using our probabilistic model (see Results), transition rates between neighbor states are given by

$q_{\left( x,y \right),\left( x,y-1 \right)}=k_{1}\frac{(2y-x)(2y-x-1)}{2}$, (A.1a)

$q_{\left( x,y \right),\left( x,y+1 \right)}=k_{2}(x-y)$, (A.1b)

$q_{\left( x,y \right),\left( x+1,y \right)}=k_{3}(2y-x)$ and (A.1c)

$q_{\left( x,y \right),\left( x-1,y \right)}=k_{4}(x-y)$. (A.1d)

Here, *q*_(_*_x_*_,_ *_y_*_),(_*_x_*_',_ *_y_*_')_ is the transition rate from a karyotype (*x*, *y*) to a neighboring karyotype (*x'*, *y'*). If the following three equations of detailed balance of a distribution of 4 states that surrounds an $1\times1$tile $(i. e.,\pi_{\left( x,y \right)}, \pi_{\left( x,y+1 \right)}, \pi_{\left( x+1,y \right)} and \pi_{\left( x+1,y+1 \right)})$,

${\pi_{\left( x,y \right)}q}_{\left( x,y \right),\left( x,y+1 \right)}={\pi_{\left( x,y+1 \right)}q}_{\left( x,y+1 \right),\left( x,y \right)}$, (A.2a)

${\pi_{\left( x,y \right)}q}_{\left( x,y \right),\left( x+1,y \right)}={\pi_{\left( x+1,y \right)}q}_{\left( x+1,y \right),\left( x,y \right)}$ and (A.2b)

${\pi_{\left( x,y+1 \right)}q}_{\left( x,y+1 \right),\left( x+1,y+1 \right)}={\pi_{\left( x+1,y+1 \right)}q}_{\left( x+1,y+1 \right),\left( x,y+1 \right)}$, (A.2c)

are satisfied, another equation of detailed balance,

${\pi_{\left( x+1,y \right)}q}_{\left( x+1,y \right),\left( x+1,y+1 \right)}={\pi_{\left( x+1,y+1 \right)}q}_{\left( x+1,y+1 \right),\left( x+1,y \right)}$, (A.2d)

hold from equation (A.1).

Then we can find π that holds the detailed balance condition in all path from (*x*, *y*) = (1, 1) to the limit. We first focus on the distributions of karyotypes with all chromosomes being acrocentric (frequency of acrocentric chromosomes *p_A_* = 100%). Using equations (A.1) and (A.2), we can obtain the following equation:

$\pi_{\left( y,y \right)}=\frac{2K_{f}}{K_{i}y}\pi_{\left( y-1,y-1 \right)}=\left( \frac{2K_{f}}{K_{i}} \right)^{y-1}\times\frac{\pi_{\left( 1,1 \right)}}{y!}$, (A.3)

where $K_{f}=\frac{k_{2}}{k_{1}}$ and $K_{i}=\frac{k_{4}}{k_{3}}$.

For the karyotype with metacentric chromosomes (the number of metacentric chromosomes = *n*_M_), using equations (A.1) and (A.2), we can obtain

$\pi_{\left( y+n_{M},y \right)}=\frac{y-n_{M}+1}{K_{i}n_{M}}\pi_{\left( y+n_{M}-1,y \right)}=\frac{{}_{y}{C_{n_{M}}}}{K_{i}^{n_{M}}}\times\pi_{\left( y,y \right)}$. (A.4)

Using equations (A.3) and (A.4),

$\pi_{\left( y+n_{M},y \right)}=\left( \frac{{2K}_{f}}{K_{i}} \right)^{y-1}\times\frac{\pi_{\left( 1,1 \right)}}{y!}\times\frac{{}_{y}{C_{n_{M}}}}{K_{i}^{n_{M}}}$. (A.5)

A distribution of chromosome number,$\pi_{y}$, is represented as

$\pi_{y}=\sum_{n_{M}=0}^{y} \pi_{\left( y+n_{M},y \right)}=\left( \frac{{2K}_{f}}{K_{i}} \right)^{y-1}\times\frac{\pi_{\left( 1,1 \right)}}{y!}\times\left( 1+\frac{1}{K_{i}} \right)^{y}=\frac{K_{i}{\pi_{\left( 1,1 \right)}\Lambda}^{y}}{2K_{f}y!}$, (A.6)

where

$\Lambda=\frac{2K_{f}(K_{i}+1)}{K_{i}^{2}}$.

Sum of the distribution is

$S=\sum_{y=1}^{\infty} \pi_{y}=\frac{K_{i}\pi_{\left( 1,1 \right)}}{{2K}_{f}}(e^{\Lambda}-1)$. (A.7)

When *S* = 1, $\pi$ is the stationary distribution. Hence,

$\pi_{\left( 1,1 \right)}=\frac{{2K}_{f}}{K_{i}(e^{\Lambda}-1)}$. (A.8)

Using equations (A.5) and (A.8), we obtain the stationary distribution

$\pi_{\left( x,y \right)}=\frac{2^{y}K_{f}^{y}{}_{y}{C_{x-y}}}{K_{i}^{x}(e^{\Lambda}-1)y!}=\frac{K_{f}^{y}}{K_{i}^{x}(e^{\Lambda}-1)(2y-x)!(x-y)!}$. (A.9)

Equations (A,6) and (A.8) gives the stationary distribution of chromosome number:

$\pi_{y}=\frac{\Lambda^{y}}{(e^{\Lambda}-1)y!}$. (A.10a)

It can be expressed as:

$\pi_{y}=\frac{1}{1-e^{-\Lambda}}\times\frac{e^{-\lambda}\Lambda^{y}}{y!}$. (A.10b)

The stationary distribution of chromosome number is the conditional probability of a Poisson distribution with mean $\Lambda$ and the condition of *y* ≥ 1.

The posterior probability of *n_M_* with a given *y* is

$\frac{\pi_{\left( y+n_{M},y \right)}}{\pi_{y}}={}_{y}{C_{n_{M}}}\left( \frac{K_{i}}{K_{i}+1} \right)^{y-n_{M}}\left( \frac{1}{K_{i}+1} \right)^{n_{M}}$. (A.11)

The probability is a binomial distribution with the number of trials = *y* and the probability of success = $\frac{1}{K_{i}+1}$.

In the stationary distribution, the expectations of *x* and *y* are

$EX(x)=\frac{K_{i}+2}{K_{i}+1}\times\frac{\Lambda}{1-e^{-\Lambda}}$, (A.12a)

$EX(y)=\frac{\Lambda}{1-e^{-\Lambda}}$. (A.12b)

The variances of *x* and *y* are

$Var(x)=\frac{\Lambda}{1-e^{-\Lambda}}\left\{ \frac{K_{i}+4}{K_{i}+1}-\left( \frac{K_{i}+2}{K_{i}+1} \right)^{2}\frac{\Lambda}{e^{\Lambda}-1} \right\}$, (A.13a)

$Var(y)=\frac{\Lambda}{1-e^{-\Lambda}}\left( 1-\frac{\Lambda}{e^{\Lambda}-1} \right)$. (A.13b)

Interestingly, the expectation of the number of metacentric chromosomes *n_M_* and the number of acrocentric chromosomes *n_A_* is simply expressed as follows:

$EX(n_{M})=EX(x-y)=\frac{1}{1-e^{-\Lambda}}\times\frac{{2K}_{f}}{K_{i}^{2}}$, (A.14a)

$EX(n_{A})=EX(2y-x)=\frac{1}{1-e^{-\Lambda}}\times\frac{{2K}_{f}}{K_{i}}$. (A.14b)

In our model, one centric fission and two A-M inversions add one metacentric chromosome to a karyotype, whereas one centric fission and one A-M inversion add one acrocentric chromosome to a karyotype. As these sequential events can be represented by the combination of the two parameters, *K_f_* and *K_i_*, which represent the ease of occurrence of centric fission and M-A transitions, respectively, these parameters determine the number of metacentric and acrocentric number in the stationary distributions. Chromosome number in the stationary distribution is given as the sum of these chromosomes. So, equation (A.12b) can be expressed as:

$EX(y)=\frac{1}{1-e^{-\Lambda}}\times\left( \frac{2K_{f}}{K_{i}^{2}}+\frac{2K_{f}}{K_{i}} \right)$. (A.12b')

The expectation of frequency of acrocentric chromosomes *p_A_* is

$EX(p_{A})=EX\left( \frac{2y-x}{y} \right)=\frac{K_{i}}{K_{i}+1}$ (A.15)

This indicates that the expected frequency of acrocentric chromosomes is independent from fusion/fission bias *K*_f_. When there is no bias in pericentric inversions (*K*_i_ = 1), the expectation of frequency of acrocentric chromosomes is constant, 0.5 in the stationary distribution.

**The mode of stationary distribution**

Here, we explore the mode of the stationary distribution. First, we only consider the case, *y* ≥ 2. Let $R_{\left( x,y \right),(x',y')}$be a ratio of probability of the state (*x*, *y*) to the state (*x’*, *y’*). Using equations (A.1) and (A.2),

$R_{\left( x,y \right),\left( x+1,y \right)}=\frac{\pi_{\left( x,y \right)}}{\pi_{\left( x+1,y \right)}}=\frac{q_{\left( x+1,y \right),(x,y)}}{q_{\left( x,y \right),(x+1,y)}}=\frac{k_{4}(x-y+1)}{k_{3}(2y-x)}$, (A.16)

where $x\leq2y-1$.

Similarly,

$R_{\left( x+1,y \right),\left( x+2,y \right)}=\frac{k_{4}(x-y+2)}{k_{3}(2y-x-1)}$, (A.17)

where $x\leq2y-2$.

Hence, using equations (A.16) and (A.17), we obtain,

$R_{\left( x+1,y \right),\left( x+2,y \right)}=\frac{x-y+2}{x-y+1}\times\frac{2y-x}{2y-x-1}\times R_{\left( x,y \right),\left( x+1,y \right)}>R_{\left( x,y \right),\left( x+1,y \right)}$, (A.18)

where $x\leq2y-2$.

Then, the inequality,

$R_{\left( x,y \right),(x+1,y)}=\frac{K_{i}\left( x-y+1 \right)}{2y-x}\geq1$, (A.19)

gives the condition that $\pi_{\left( x,y \right)}$ is equivalent or higher than the probability of the right states along the *x*-axis. Similarly, the inequality,

$R_{\left( x,y \right),(x-1,y)}=\frac{2y-x+1}{K_{i}(x-y)}\geq1$, (A.20)

gives the condition that $\pi_{\left( x,y \right)}$ is equivalent or higher than the probability of the left states along the *x*-axis. Therefore, the karyotype (*x*, *y*) that satisfies both inequalities (A.19) and (A. 20) is the mode of the stationary distribution in a given chromosome number *y*. If the arm number at the mode of the stationary distribution with a given chromosome number *y* is denoted by *x^*^*(*y*), the range of *x*^*^(*y*) is given as follows, using inequaliies (A.19) and (A.20);

$\frac{K_{i}+2}{K_{i}+1}y+\frac{1}{K_{i}+1}\geq x^{*}(y)\geq\frac{K_{i}+2}{K_{i}+1}y-\frac{1}{K_{i}+1}$. (A.21)

The range of difference in *x*^*^ between *x*^*^(*y*) and *x*^*^(*y* + 1) is calculated as follows;

$2+\frac{1}{K_{i}+1}>x^{*}(y+1)-x^{*}(y)>\frac{1}{K_{i}+1}$. (A.22)

Because *x*^*^ is an integer,

$x^{*}\left( y\pm1 \right)=x^{*}\left( y \right)\pm1 orx^{*}\left( y \right)\pm2$, where double-sign corresponds. (A. 23)

Similarly to the *x*-axis, the two inequalities along the *y*-axis

$R_{\left( x*\left( y \right),y \right),(x*(y+1),y+1)}\geq1$ (A.24a)

$R_{\left( x*\left( y \right),y \right),(x*(y-1),y-1)}\geq1$ (A.24b)

give the condition that (*x*^*^(*y*), *y*) is the mode of the stationary distribution. Each inequality of (A.24) is expressed as two inequalities given equation (A.23). Then, the inequalities

$R_{\left( x*\left( y \right),y \right),(x*\left( y \right)+1,y+1)}=\frac{K_{i}\{2y-x^{*}(y)+1\}}{2K_{f}}\geq1$, (A.25a)

$R_{\left( x*\left( y \right),y \right),(x*\left( y \right)+2,y+1)}=\frac{K_{i}^{2}\left\{ x^{*}\left( y \right)-y+1 \right\}}{2K_{f}}\geq1$, (A.25b)

$R_{\left( x*\left( y \right),y \right),(x*\left( y \right)-1,y-1)}=\frac{2K_{f}}{K_{i}\{2y-x^{*}(y)\}}\geq1$, and (A.25c)

$R_{\left( x*\left( y \right),y \right),(x*\left( y \right)-2,y-1)}=\frac{2K_{f}}{K_{i}^{2}\{x^{*}(y)-y\}}\geq1$, (A.25d)

give the condition of the stationary distribution.

Therefore, from inequalities (A. 19), (A. 20) and (A. 25), the condition can be expressed as:

$\frac{K_{i}(x-y+1)}{2y-x}\geq1$, (A.26a)

$\frac{2y-x+1}{K_{i}(x-y)}\geq1$, (A.26b)

$\frac{K_{i}(2y-x+1)}{2K_{f}}\geq1$, (A.26c)

$\frac{K_{i}^{2}(x-y+1)}{{2K}_{f}}\geq1$, (A.26d)

$\frac{{2K}_{f}}{K_{i}(2y-x)}\geq1$ and (A.26e)

$\frac{{2K}_{f}}{K_{i}^{2}(x-y)}\geq1$. (A.26f)

The condition (A.26) can be also expressed as:

$1\geq K_{i}\left( x-y \right)-\left( 2y-x \right)\geq{-K}_{i}$, (A.27a)

$\frac{2K_{f}}{K_{i}}\geq2y-x\geq\frac{2K_{f}}{K_{i}}-1$ and (A.27b)

$\frac{2K_{f}}{K_{i}^{2}}\geq x-y\geq\frac{2K_{f}}{K_{i}^{2}}-1$, (A.27c)

If *x* is equal to *y* (≥ 2) (i.e., *p_A_* = 100%) in the condition (A.27), the condition is expressed as

$K_{i}\geq2$ and (A.29a)

$\frac{{K_{i}}^{2}}{2}\geq K_{f} \geq K_{i}$. (A.29b)

And the range of *y* is

$\frac{2K_{f}}{K_{i}}\geq y\geq\frac{2K_{f}}{K_{i}}-1$. (A.30)

The condition for the mode at a karyotype with *p*_A_ = 100% is given by large *K*_f_ and *K*_i_. If *x* is equal to 2*y* (≥ 4) (i.e.,  *p*_A_ = 0%) in the condition (A.27), the condition is expressed as

$K_{i}\leq0.5$and (A.31a)

$\frac{K_{i}}{2}\geq K_{f} \geq{K_{i}}^{2}$. (A.31b)

And the range of *y* is

$\frac{2K_{f}}{K_{i}^{2}}\geq y\geq\frac{2K_{f}}{K_{i}^{2}}-1$. (A.32)

The condition for the mode at a karyotype with *p_A_* = 0% is given by small *K_f_* and *K_i_*. Numerical examples of the mode of the stationary distributions were shown in Fig A1 (see the end of S1 Appendix). We finally consider the case with *y* = 1. A condition for the mode at (*x*, *y*) = (1, 1) is given by the inequalities,

$R_{\left( 1,1 \right),(1,2)}\geq1$ and (A.33a)

$R_{\left( 1,1 \right),(x*(2),2)}\geq1$. (A.33b)

This is simply expressed as

$K_{i}\geq1$, (A.34a)

$K_{i}\geq K_{f}$ and (A.34b)

$\frac{{K_{i}}^{2}}{2}\geq K_{f}$. (A.34c)

Similarly, a condition for the mode at (*x*, *y*) = (2, 1) is given by the inequalities,

$K_{i}\leq1$, (A.35a)

$\frac{K_{i}}{2}\geq K_{f}$ and (A.35b)

${K_{i}}^{2}\geq K_{f}$. (A.35c)

These suggest that relatively small *K_f_* gives the mode at *y* = 1.

**Model with the arbitral limitation of chromosome number**

For application of our model, we used a definite space in the karyograph with arbitrarily determined maximum limits of chromosome number *y*_max_. Under this model with a definite space, we assumed the same transition rates except

$q_{\left( x,y_{max} \right),\left( x,y_{max}+1 \right)}=0$. (A.36)

In this case, the stationary distribution is expressed as

${\pi'}_{(x,y)}=\frac{K_{f}^{y}}{\left( \sum_{y=1}^{y_{max}} \frac{\Lambda^{y}}{y!} \right)K_{i}^{x}(x-y)!(2y-x)!}$. (A.37)

The ratio of stationary distribution of this model (A.37) to that of the model with infinite space (A.9) is constant:

$\frac{{\pi'}_{(x,y)}}{\pi_{(x,y)}}=\frac{e^{\Lambda}-1}{\sum_{y=1}^{y_{max}} \frac{\Lambda^{y}}{y!}}$. (A.38)

This indicates that the use of the definite space doesn’t change the relative frequency of different karyotype states in the given space. In our application, we empirically validated the effect of *y*_max_ by comparing the parameter estimates between different values of *y*_max_ (*y*_max_ = 30 and *y*_max_ = 35; S5 Table). Although *k*_2_ in Otophysi was affected quantitively, it didn’t affect our conclusion qualitatively.

**Fig A1** **Numerical examples of the mode in stationary distribution.** Blue, red, and green lines indicate boundaries in the condition (A.25a), (A.25b), and (A.25c), respectively (see S1 Appendix). Enclosed points demonstrate the modes of the stationary distributions with given *K*_f_ and *K*_i_. (A) *K*_f_ = 6.25 and *K*_i_ = 1, (B) *K*_f_ = 288 and *K*_i_ = 24, (C) *K*_f_ = 0.364 and *K*_i_ = 0.182, (D) *K*_f_ = 0.019 and *K*_i_ = 0.037. B, C, and D are the cases when the mode is located at the local maxima of fish; **(24, 24)**, **(47, 25),** and **(54, 27)**, respectively.
