# Supplementary figures and images for: Tempo and mode in karyotype evolution revealed by a probabilistic model incorporating both chromosome number and morphology
Source: PLoS Genet. 2021 Apr 16;17(4):e1009502. doi: 10.1371/journal.pgen.1009502 (PMC8081341; doi:10.1371/journal.pgen.1009502)

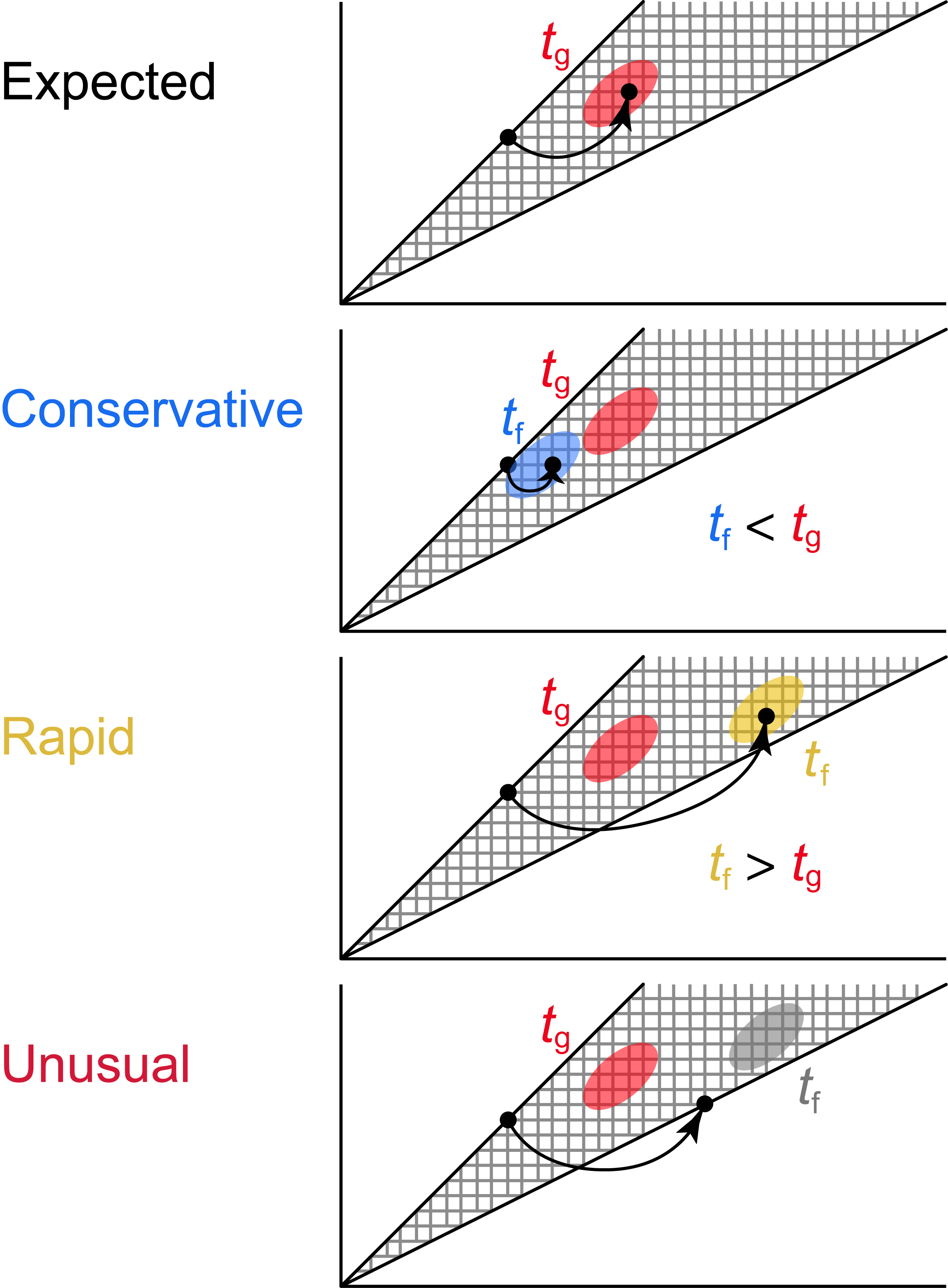

Supplement: S14 Fig — The filled circles indicate expected ranges (99%). When the karyotype after the transition is within the range expected from tg, the branch is categorized as "Expected." When the karyotype after the transition is out of the range expected from tg but within the range expected from tf, the branch is categorized as "Conservative" (tg > tf) or "Rapid" (tg < tf). When the karyotype after the transition is out of the range expected from both tg and tf, the branch is categorized as "Unusual." tg, actual time estimated from the phylogenetic tree; tf, fitted time of the branch. (TIF) [file pgen.1009502.s015.tif]
