# Supplementary material for: Tempo and mode in karyotype evolution revealed by a probabilistic model incorporating both chromosome number and morphology
Source: PLoS Genet. 2021 Apr 16;17(4):e1009502. doi: 10.1371/journal.pgen.1009502 (PMC8081341; doi:10.1371/journal.pgen.1009502)
Supplement: Attachment — Submitted filename: PLOS_G_Response to reviewers2.pdf [file pgen.1009502.s026.pdf]

This manuscript has been carefully evaluated by two referees. Each is enthusiastic about the topic and modeling approach. Both raise key questions about the generality of the method and offer suggestions for comparisons to other modeling approaches and for testing model performance with simulated data. Relatedly, the reviewers seek additional details for how the data selection procedure might have influenced the findings. Given the potential high level of interest in this work and the potential to apply this model to other taxa, the manuscript would benefit from additional attention to these points and others raised in the reviews.

Response: Thank you very much for handling our paper and giving us excellent feedbacks. We are glad to hear that both reviewers are "enthusiastic about the topic and modeling approach". We followed suggestions made by the reviewers and have revised the manuscript. The parts changed during revision are highlighted in red letters in the manuscript. We also made several minor edits to increase the clarity (but not to change the content), which are also highlighted in red letters.

The major revisions that we made can be summarized as follows.

1) Addition of simulations: As suggested by Reviewer #2, we have conducted several simulations to evaluate our methods. To this end, we replaced MuSSE for Mk-n in the revised paper. In the original manuscript, we used Mk-n, but it was much slower than MuSSE (both are implemented in diversitree R package). Therefore, we used MuSSE throughout the revised paper. Before replacing MuSSE for Mk-n, we confirmed that both methods gave rise to very similar results (see the text). During revisions, we found an error in the original script that set an unnecessary constraint, and we have corrected it. This correction increased the range of the estimated ancestral karyotype state, and the certainty of the ancestral state of Otophysii has reduced. So, we softened the tone about the directional evolution of karyotype in Otophysii. Overall, the results were qualitatively similar.
2) Analysis of the influence of data selection: To test whether the exclusion of several species based on the maximum chromosome number and polyploidization influenced our results, we conducted analyses using a clade that does not contain any species that were filtered out. We confirmed that the same conclusions regarding the biased transitions and differences in the extinction rates among karyotypes were obtained (see below).
3) Comparison with synteny-based analysis: As suggested by Reviewer #1, we compared our results on the estimate of ancestral karyotype with those of synteny-based approach in Discussion. We also discussed both merits and demerits of both methods and clarified that our method is complementary to the synteny-based analysis.

4) Effect of polyploidy: We made a model including polyploidization and applied that model to a plant group Brassicaceae.

Reviewer's Responses to Questions

Comments to the Authors:
Please note here if the review is uploaded as an attachment.

Reviewer #1: Yoshida and Kitano present a comprehensive manuscript regarding karyotype evolution reconstruction. Indeed, this is an important and intriguing topic in evolutionary genomics allowing the understanding of how evolution shaped the modern karyotypes in line with adaptation and speciation. In this context, the authors built an original probabilistic model of karyotype evolution incorporating both chromosome and arm numbers, in order to reconstruct the karyotype evolution from common ancestors to modern species in terms of chromosome (numbers and types) and arm numbers. Then, they applied the established model to two major fish taxa, Eurypterygii, and series Otophysi. With this analysis, the authors identified that Eurypterygii species exhibit a conservative karyotype evolution with a high extinction rate in species with a different karyotype while the karyotype evolved in the same direction in multiple lineages of Otophysi making the most common extant karyotype different from the ancestral state.

Overall, I find this manuscript clear and interesting, and such a probabilistic model of karyotype evolution taking into consideration chromosome and arm numbers is indeed novel and represents an asset for karyotype evolution studies. However, I think that some important aspects are still missing in the model, it seems necessary to me to apply it to synteny-based karyotype evolution analyses and reconstructions in plants and/or vertebrates from the literature. It is great to apply the model to two large taxonomic groups of fish and the obtained results are meaningful but don’t look sufficient to assess the robustness of the model and don’t represent significant advances in our understanding of genetic and biological mechanisms. For these reasons, I would classify this manuscript as a method article rather than an original research. Moreover, it still needs to be applied to other taxa and compared with synteny-based analyses, hence the following major issues.

Major issues:

-It would be crucial to apply the established model to other group of species (plants and/or

vertebrates) for which synteny-based approaches have been applied to study the karyotype evolution from reconstructed ancestors to the modern species. Indeed, it would be crucial to check whether similar results are obtained and how complementary those two approaches are. For instance, this kind of analysis has been performed in cereals (Murat et al. 2014, GBE) and vertebrates (Simakov et al. 2020, Nature Ecology and Evolution; Nakatani et al. 2007, Genome Research).

Response: Thank you so much for excellent suggestions. We agree that synteny-based analysis and our model-based approaches are complementary. To compare with synteny-based analysis, we applied our model to a plant group Brassicaceae, whose karyotype evolution has been extensively investigated using the synteny-based approach. We reached conclusions similar to those of previous synteny-based analysis in that the ancestral chromosome number would be 7-10, and several extant species with smaller numbers of chromosomes are derived from fusions. We added these analyses in the Results (L371-390). We also made a new paragraph to discuss the consistency of our results in fish and plant species with previous synteny-based inferences of these taxa and the merits and demerits of both approaches (L416-342).

-Whole genome duplications (WGD) are a recurrent and major source of karyotype plasticity during plant evolution and also occurred during vertebrate evolution (1R, 2R, 3R). This mechanism impacts dramatically the karyotype during evolution by doubling the chromosome number and has been shown to be followed by extensive chromosomal rearrangements (chromosome number reduction). This parameter should be taken into consideration in the model given that such a mechanism would dramatically bias the outcome of it.

Response: In fishes, polyploidization events are well described and rarer compared to plants. That is one of the reasons why we focused on the fishes in this paper. However, we agree with the reviewers that polyploidization should be taken into consideration when applying this method to other taxa in the future. Therefore, we made a model incorporating the polyploidization rate as a parameter, denoted as k_5, and applied that model to a plant group Brassicaceae. We could successfully infer all parameters, including the polyploidization rate. We also compared the results obtained with all 39 species with those after excluding previously reported polyploid species (10 species). Both analyses gave rise to similar results (L371-390).

Unfortunately, we could not apply the model with polyploidization to fishes, because the maximum of reported chromosome number in fishes is 75 and we should set the limit of chromosome number (y_{max}) to a much higher value than that used in the present study, which needs a far higher computational power. We admit that there is room for further improvement in the programming. We discuss it in the Discussion (L437-448).

Minor issues:

-Particular attention could be paid to the figure design. For instance, Figure 3 and Figure 4 look very raw and the article would benefit from more sophisticated figures.

Response: We revised the figures (see Fig 3 and Fig 4).

-The synteny-based approaches for karyotype evolution studies should be mentioned and referenced.

Response: We agree that synteny-based analysis and our model-based approaches are complementary. In Discussion, we discussed this point as explained above.

Reviewer #2: This paper develops and applies a new model for the evolution of genome structure (chromosome number and arm number) and applies it to two fish groups. I think this paper is, on the whole, a great contribution and that the findings are broadly interesting. I believe a few things should probably be explored a bit more, which is discussed below.

Major Comments

line 34-37 (and other parts of the manuscript):

The ending of the abstract and the paper as a whole makes a bold inference with regard to the finding that higher extinction rates are associated with rare karyotypes. However, there is no discussion of the well-documented problem of false positives with this type of SSE approach.

(Rabosky and Goldberg 2015, Louca and Pennell 2020, etc.)

Response: Thank you very much for pointing this out. First, we softened the tone about the conclusions drawn from the SSE approach in Abstract (L36, L483-485) and Discussion. We also cited these papers and discussed possible issues of our SSE analyses in Discussion (L499-502): “Finally, although we found an association between karyotype and speciation/extinction rates in multiple taxa, it should be noted that binary-state speciation and extinction models can produce false positive results (48, 49), so our results should be interpreted with caution.”

It should be noted that Rabosky and Goldberg (2015) pointed out the importance of replication across clades. We have followed this suggestion and applied the SSE approach to two independent fish lineages and also subclades of Eurypterygii.

I am also curious whether or not the data selection process could be driving this result. In lines 413-415, you describe your selection process; however, you do not report the number of records discarded due to these heuristic selection rules. Likewise, on line 469 you say that you didn't use species with chromosome number greater than 30. How many taxa does this remove from your dataset? Isn't it possible that this thinning of records, particularly extreme ones, could lead to an association with a signal for extinction and extreme karyotypes?

Response: First, we clarified the number of species that were filtered out in the Materials and Methods (L512-515). Second, to examine the effects of data filtering on our results, we conducted the analyses using a subgroup of Eurypterygii, series Eupercaria, which have no species filtered out. Even without any data filtering, we reached the same conclusion that the extinction rates increased in species with karyotypes different from the mode. We described the results in L309-317: “As three species of Eurypterygii were filtered out in our analysis due to the large chromosome number ($y > 35$) or polyploidy, we examined whether this filtering biased our results. A subgroup of Eurypterygii, series Eupercaria, included no species that were filtered out. The results of the analysis using only Eurpercaria showed that the extinction rate was much lower in the karyotype **(24, 24)**, although the speciation rate was also slightly lower in the karyotype **(24, 24)** than in other karyotypes (S2 Table). The model with

variable speciation and extinction rates was again more highly supported than the model with constant speciation and extinction rates (likelihood ratio test, *p*-value $< 2.2\times10^{-16}$, S2 Table)."

I think there are several things that you could do to get around these problems. One avenue would be to simulate karyographs with your model and inferred parameters on the empirical tree and ask how often you see a result as extreme as what you observe. This would seem to tell you whether you perhaps have a small part of your tree with a strong signal and that any karyotype inferred to be present at that point in the tree will be associated with high extinction rates. Alternatively, you could find that the signal for high extinction rates is supported be many subtrees in your phylogeny and you rarely see a signal as strong as you observe in your data.

Response: First, we conducted simulations of karyograph using inferred parameters and the empirical tree as suggested. Without inclusion of speciation and extinction rates, we found that all of 10,000 simulations indicated that karyotypes were more dispersed than the empirical data. This result is consistent with our hypothesis that species with karyotype other than the mode have higher extinction rates (see Results in L282-288). Second, we conducted the analyses of speciation/extinction rates using not only all Eurypterygii but also subgroups of this group (L309-317). We also conducted the same analysis in Otophysi (L318-325). In all analyses, we found that karyotype is associated with extinction.

Another possible route to confirm or refute this would be to fit a model like BAMM on the full fish tree and your pruned tree. If the rates estimated in the regions where you find higher extinction rates remain similar on the two trees, then your sampling process is unlikely to be causative in your finding.

Response: We conducted our analysis using a subgroup Eurypterygii, Eupercaria, and an independent group, series Otophysi, and reached the same conclusion (see above).

More broadly, some assessment of the amount of your tree and the number of independent regions of your phylogeny leading to this inference is necessary. What is the effective sample size that is driving this result? By this I don't mean a specific number but how many portions of

your tree are supporting this association (i.e. Maddison and Fitzjohn 2015).

Response: We conducted simulations with different numbers of species and showed how the sample sizes influence the certainty and range of the estimated parameters (L203-227).

Methods section:

Since this is a brand new model of trait evolution, it would be nice to see some assessment of how well it works with simulated data and how well it captures the key aspects of trait evolution in your particular dataset. Perhaps both of these could be accomplished with a posterior predictive simulation (PPS) analysis where you simulate new karyotypes based on the inferred rate parameters and ancestral state inferred at the tree's base. For each of the simulated datasets you could evaluate how well you do at inferring the now known ancestral states as well as the parameters of your model. I think this is essential to know if there are any aspects of the model performance to be concerned with or if certain parameters are difficult to estimate or biased in certain ways.

Response: Following this excellent suggestion, we have conducted these simulations (L179-227). We added results of simulations in the Results. When the analyzed clade is smaller, the certainty of the parameters reduces, although more recent ancestral nodes have higher certainty of ancestral state reconstruction.

Line 334: You say that you exclude polyploid species from your analysis. How was this done? Polyploid species often do not have a clear doubling of chromosome number. I think including the number of species excluded from each clade based on these decisions is important. This issue ties in with another concern that your model doesn't allow for chromosome number changes through ascending disploidy. The PPS analysis suggested above might reveal whether this is anything to worry about. For instance, if it is common in your dataset, your simulated datasets will likely show a different chromosome/arm number distribution relative to your empirical dataset.

Response: In fishes, polyploidization events are well described and rarer compared to plants. Arai (2011) described which species are polyploid and we followed that description. That is one

of the reasons why we focused on the fishes in this paper. However, we agree with the reviewers that polyploidization should be taken into consideration when applying this method to some other taxa in the future. Therefore, we made a model incorporating the polyploidization rate as a parameter and applied that model to a plant group Brassicaceae (L372-390). See Response to Reviewer #1 above.

We agree that chromosome number change is not only due to centric fission and fusion, but can be cause by telomere fusion or non-centric fission. These are generally deleterious, so we did not consider these types of karyotype changes in the present study, but we mention these possibilities in Discussion (L457-464): “Finally, we assumed that the change in chromosome number occurs via centric fusion or fission. However, chromosome number can change by non-centric mechanisms, such as telomere fusion and non-centric fission. Telomere fusion can generate a dicentric chromosome, which can be deleterious (37). As non-centric fission splits one chromosome into two, with only one having a centromere and the other lacking a centromere, it can have deleterious effects (38). Therefore, we did not consider these types of fusion and fission. When some taxa, however, have higher rates of this type of karyotype evolution, these rates should also be included as parameters.”

Minor Comments:

line 42-43: I think that it is inaccurate to say that we don't know if the karyotype itself has any functional role. It seems that a great deal of work has been done considering how chromosome number and the division of the genome into autosomes and sex chromosomes impacts: evolution of certain genetic architectures, geneflow, recombination, and reproductive barriers.

Kitano, J., Ross, J.A., Mori, S., Kume, M., Jones, F.C., Chan, Y.F., Absher, D.M., Grimwood, J., Schmutz, J., Myers, R.M. and Kingsley, D.M., 2009. A role for a neo-sex chromosome in stickleback speciation. Nature, 461(7267), pp.1079-1083.

Anderson, N.W. and Blackmon, H., 2020. The Probability of Fusions Joining Sex Chromosomes and Autosomes. bioRxiv.

Response: Following this advice, we changed this sentence to “we have not reached a consensus on how rapidly karyotype can evolve and whether a particular karyotype is favored.” (L41-43)

line 60-61 I would say that heterokaryotypic individuals experience the full range of fertility effects for instance:

Ratomponirina C, Brun B, Rumpler Y. Kew Chromosome Conference III: Synaptonemal complexes in Robertsonian translocation heterozygous in lemurs.; 1988. p. 65–73

In this study, a range of crosses were made with varying degrees of difference in chromosome number, and spermatogenesis was studied in each F1.

Response: We agree that heterokaryotypic hybrids can show normal fertility. We changed this sentence to “For instance, interbreeding of individuals with different karyotypes results in heterokaryotypic hybrids with impaired fertility, although there are cases that heterokaryotypes have normal fertility (4).” (L59-62)

Line 198: I would remind your reader what k1-4 are at this point, it is a lot to keep it in their head and not have to flip back and forth.

Response: We explained four parameters here: “four evolutionary parameters (k_1, fusion rate coefficient; k_2, fission rate coefficient; k_3, A-M transition rate coefficient; k_4, M-A transition rate coefficient).” (L250-252)

Line 234-241: It would be good to include some measures of certainty in the estimates that you report in this paragraph and elsewhere. Saying the most probable is 24 is at best an incomplete picture it could be with 99.9% certainty or all values between 10 and 30 could have nearly identical support, and 24 is just .1% more probable.

Response: We added the information of 95% range wherever possible.

Line 506-507: Since diversitree has the sampling proportion approach built into it to deal with sampling biases, why did you not use it? Is your method better or equivalent in performance? If possible, give a citation and or an explanation as to why this choice was made.

Response: Thank you for pointing this out. We agree that the correction method using the sampling fraction, which is suggested by Reviewer #2, is more suitable for comparing speciation and extinction rates between karyotypes. Therefore, we re-analyzed the data using this correction method in the revised manuscript (see Materials and Methods in L62-631).

Finally, we would like to thank all reviewers and editors for giving us excellent feedbacks.
